# Journal response time: an experience at Pan African Medical Journal

**DOI:** 10.11604/pamj.2026.53.133.49254

**Published:** 2026-03-17

**Authors:** Gina Joubert

**Affiliations:** 1 Department of Biostatistics, University of the Free State, Bloemfontein, South Africa

**Keywords:** Journal response time, comment, review, communication, Pan African Medical Journal

## Abstract

This comment outlines the author´s experience with a submission to the Pan African Medical Journal. Twenty-two months after submission, the manuscript remains in review, and no first decision from the journal has been received. The author urges the editorial office to put measures in place to ensure that other authors do not have a similar experience.

## Commentary

In 2019, colleagues and I published a study in the Pan African Medical Journal (PAMJ) regarding journal response times [1]. Our findings showed that the median number of days from submission to first journal response was 15 days for rejected manuscripts (range 0-381 days), 48 days for resubmissions (range 27-346 days), and 81 days for manuscripts requiring revisions (range 18-546 days) [1]. We stated that “the extreme isolated first response times of more than a year are ethically unacceptable since during that time the authors cannot submit the material elsewhere, and by the time the journal does respond with a rejection, the findings and literature may be outdated” [1].

In May 2024, I submitted a short communication to PAMJ, which was acknowledged by email on 16^th^ May, 2024. The journal website indicates that a first response is, on average, after 6 weeks, and one should not enquire before that time has elapsed. I have enquired regarding the manuscript´s progress three times. My first enquiry in August 2024 was responded to in October 2024, and I was informed that the manuscript was still with a reviewer. My second enquiry in May 2025 (a year after submission) was not responded to. On 8^th^ September 2025, I enquired again (15 months after submission) and received a rapid response that was similar to the one I received in October 2024. On the journal platform, the last action listed for this manuscript indicated that it was assigned to an editor late in May 2024, and the current status is that it is under review. Compared to the findings of our previous study, the time that has elapsed since the submission is already longer than the maximum for rejected manuscripts and resubmissions. I can only hope that this bodes well for receiving feedback (soon!), indicating that revision is required.

My experience that 22 months have elapsed since submission without a first decision being made by the journal, with the submission remaining in review, may be an isolated and unusual occurrence at PAMJ, but I urge the editorial office to put measures in place to ensure that other authors do not have a similar experience.
